# Supplementary material for: Viviparity imparts a macroevolutionary signature of ecological opportunity in the body size of female Liolaemus lizards
Source: Nat Commun. 2024 Jun 11;15:4966. doi: 10.1038/s41467-024-49464-x (PMC11167029; doi:10.1038/s41467-024-49464-x)
Supplement: Supplementary file 1 — Supplementary Information [file 41467_2024_49464_MOESM1_ESM.pdf]

Supplementary information

Viviparity imparts a macroevolutionary signature of ecological opportunity in the body size of female *Liolaemus* lizards

Saúl F. Domínguez-Guerrero[1*], Damien Esquerré[2], Edward D. Burress[1,3], Carlos A. Maciel-Mata[4], Laura R. V. Alencar[1], and Martha M. Muñoz[1].

This document includes:

Supplementary Figures……………………………………………………………………2

Supplementary Figure 1…………………………………………………………...2

Supplementary Figure 2……………………………………………………………3

Supplementary Tables…………………………………………………………………...4

Supplementary Table 1…………………………………………………………….4

Supplementary Table 2…………………………………………………………….4

Supplementary Table 3…………………………………………………………….5

Supplementary Table 4…………………………………………………………….5

Supplementary Table 5…………………………………………………………….6

Supplementary Table 6…………………………………………………………….6

Supplementary Table 7…………………………………………………………….7

Supplementary Table 8…………………………………………………………….8

Supplementary Table 9…………………………………………………………….9

Supplementary Table 10…………………………………………………………..10

Supplementary Table 11…………………………………………………………..10

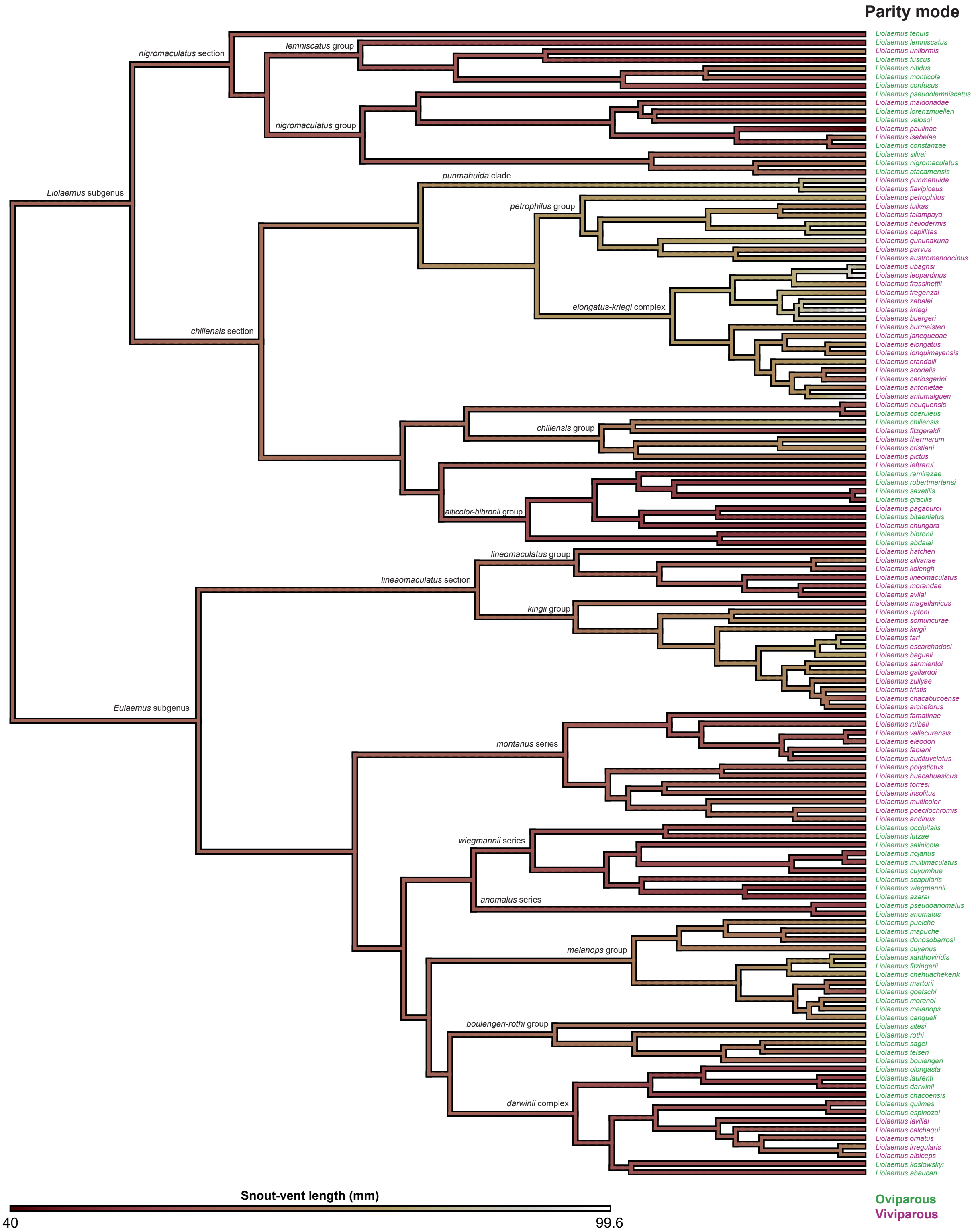

Supplementary Figure 1. Trait mapping of body size (snout-to-vent length, mm) in female *Liolaemus* lizards. Source data are provided as a Source Data file.

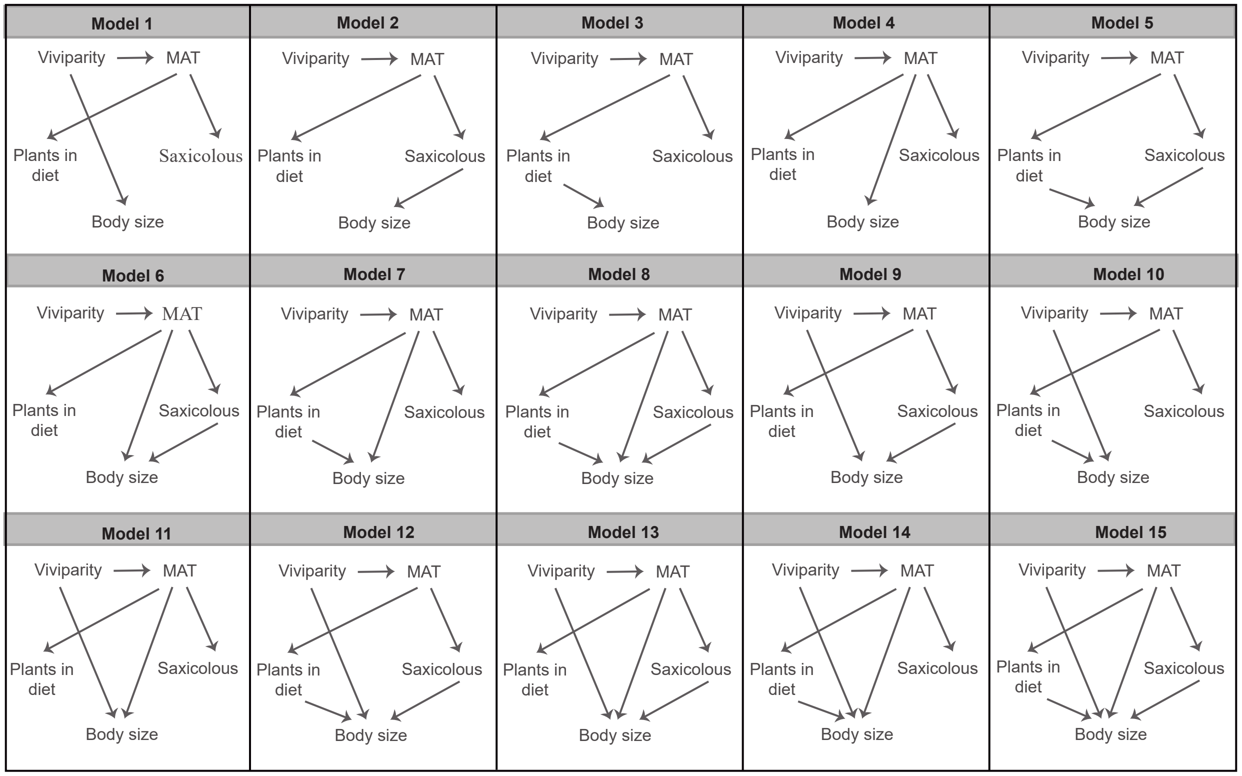

Supplementary Figure 2. Fifteen models describing the direct (Model 1), indirect (Model 2-8), and direct/indirect (Model 9-15) effects of viviparity on body size in *Liolaemus* lizards. The direct model suggests that viviparity is intrinsically associated with body size, the indirect models suggest that viviparity is extrinsically associated with body size, and the direct/indirect models suggest that viviparity is intrinsically and extrinsically associated with body size. These models were simultaneously fitted and compared using phylogenetic path analysis (see Methods and Results). MAT= Mean annual temperature.

Supplementary Table 1. Summary of five model fits for the body size (snout-to-vent length; SVL) data across 500 stochastic character maps of parity mode through the maximum clade credibility tree. A brief description of each model is as follows: (1) BM1: a single-rate model of stochastic trait evolution (*i.e.*, rate constrained to be the same for viviparous and oviparous species), (2) BMS: a two-rate model of stochastic trait evolution (*i.e.*, separate rates for viviparous and oviparous species), (3) OU1: a single phenotypic optimum (*i.e.*, shared optimum for viviparous and oviparous species) and a single-rate model, (4) OUM: a two optima, single-rate model, and (5) OUMV: a two optima, two-rate model. This analysis was conducted with SVL data from 133 species (58 oviparous and 75 viviparous). For the best-fitting models (equivalent support, $\Delta AICc \leq 2$), we provide the rate of trait evolution (σ^2), the strength of selection (α), and the phenotypic optimum (θ). As the phenotypic optimum values are similar in the best supported models (OUM and OUMV), we focused on results from the least-complex model (OUM).

**Model**	**ΔAICc**	**Weight**	**Rate of trait evolution (σ^2) and alpha (α)**	**Phenotypic optimum (θ)**
BM1	27.33	<0.001	-	-
BMS	23.34	<0.001	-	-
OU1	5.6	0.03	-	-
**OUM**	**0**	**0.56**	**σ^2=0.0016 and α=0.142 for all species**	**56 mm SVL for oviparous species and 66.1 mm SVL for viviparous species**
OUMV	0.67	0.4	σ^2= 0.0013 for oviparous species, 0.0018 for viviparous species, and α= 0.132 for all species	55.9 mm SVL for oviparous species and 66.2 mm SVL for viviparous species

Supplementary Table 2. Summary of five model fits for the body size (snout-to-vent length; SVL) data across 500 stochastic character maps of diet through the maximum clade credibility tree. A brief description of each model is as follows: (1) BM1: a single-rate model of stochastic trait evolution (*i.e.*, rate constrained to be the same for insectivorous and omnivorous/herbivorous species), (2) BMS: a two-rate model of stochastic trait evolution (*i.e.*, separate rates for insectivorous and omnivorous/herbivorous species), (3) OU1: a single phenotypic optimum (*i.e.*, shared optimum for insectivorous and omnivorous/herbivorous species) and a single-rate model, (4) OUM: a two optima, single-rate model, and (5) OUMV: a two optima, two-rate model. This analysis was conducted with SVL data from 119 species (64 insectivorous and 55 omnivorous/herbivorous species). For the best-fitting models (equivalent support, $\Delta AICc \leq 2$), we provide the rate of trait evolution (σ^2), the strength of selection (α), and the phenotypic optimum (θ). We focused on results from the least-complex model (OU1).

**Model**	**ΔAICc**	**Weight**	**Rate of trait evolution (σ^2) and alpha (α)**	**Phenotypic optimum (θ)**
BM1	15.58	<0.001	-	-
BMS	14.93	<0.001	-	-
**OU1**	**0.97**	**0.27**	**σ^2=0.0013 and α=0.095 for all species**	**60.59 mm SVL for all species**
OUM	0.8	0.29	σ^2=0.0013 and α=0.1 for all species	57.45 mm SVL for insectivorous species and 64.43 mm SVL for omnivorous/herbivorous species
OUMV	0	0.44	σ^2=0.0016 for insectivorous species, 0.0009 for omnivorous/herbivorous species, and α=0.1 for all species	57.32 mm SVL for insectivorous species and 64.55 mm SVL for omnivorous/herbivorous species

Supplementary Table 3. Summary of five model fits for the body size (snout vent length; SVL) data across 500 stochastic character maps of substrate use through the maximum clade credibility tree. A brief description of each model is as follows: (1) BM1: a single-rate model of stochastic trait evolution (*i.e*., rate constrained to be the same for terrestrial and saxicolous species), (2) BMS: a two-rate model of stochastic trait evolution (*i.e*., separate rates for terrestrial and saxicolous species), (3) OU1: a single phenotypic optimum (*i.e*., shared optimum for terrestrial and saxicolous species) and a single-rate model, (4) OUM: a two optima, single-rate model, and (5) OUMV: a two optima, two-rate model. This analysis was conducted with SVL data from 123 species (63 terrestrial and 60 saxicolous). For the best-fitting model (OUMV), we provide the rate of trait evolution (σ^2), the strength of selection (α), and the phenotypic optimum (θ).

Model	ΔAICc	Weight	Rate of trait evolution (σ^2) and alpha (α)	Phenotypic optimum (θ)
BM1	27.8	<0.001	-	-
BMS	16.1	<0.001	-	-
OU1	8.7	0.01	-	-
OUM	6.2	0.04	-	-
**OUMV**	**0**	**0.95**	**σ^2= 0.0019 for saxicolous species, 0.0008 for terrestrial species, and α=0.108 for all species**	**66.3 mm SVL for saxicolous species and 57.3 mm SVL for terrestrial species**

Supplementary Table 4. Summary of five model fits for the body size (snout-to-vent length; SVL) data across 500 randomly-sampled trees from the posterior distribution (one stochastic character map of parity mode for each tree). A brief description of each model is as follows: (1) BM1: a single-rate model of stochastic trait evolution (*i.e*., rate constrained to be the same for viviparous and oviparous species), (2) BMS: a two-rate model of stochastic trait evolution (*i.e*., separate rates for viviparous and oviparous species), (3) OU1: a single phenotypic optimum (*i.e*., shared optimum for viviparous and oviparous species) and a single-rate model, (4) OUM: a two optima, single-rate model, and (5) OUMV: a two optima, two-rate model. This analysis was conducted with SVL data from 133 species (58 oviparous and 75 viviparous). For the best-fitting models (equivalent support, $\Delta AICc \leq 2$), we provide the rate of trait evolution (σ^2), the strength of selection (α), and the phenotypic optimum (θ). As the phenotypic optimum values are similar in the best supported models (OUM and OUMV), we focused on results from the least-complex model (OUM).

Model	ΔAICc	Weight	Rate of trait evolution (σ^2) and alpha (α)	Phenotypic optimum (θ)
BM1	28.3	<0.001	-	-
BMS	24.3	<0.001	-	-
OU1	5.6	0.034	-	-
**OUM**	**0**	**0.55**	**σ^2= 0.0016 and α= 0.143 for all species**	**55.9 mm SVL for oviparous species and 66.2 mm SVL for viviparous species**
OUMV	0.6	0.41	σ^2= 0.0013 for oviparous species, 0.0018 for viviparous species, and α= 0.133 for all species	55.9 mm SVL for oviparous species and 66.2 mm SVL for viviparous species

Supplementary Table 5. Summary of five model fits for the body size (snout-to-vent length; SVL) data across 500 randomly-sampled trees from the posterior distribution (one stochastic character map of diet for each tree). A brief description of each model is as follows: (1) BM1: a single-rate model of stochastic trait evolution (*i.e.*, rate constrained to be the same for insectivorous and omnivorous/herbivorous species), (2) BMS: a two-rate model of stochastic trait evolution (*i.e.*, separate rates for insectivorous and omnivorous/herbivorous species), (3) OU1: a single phenotypic optimum (*i.e.*, shared optimum for insectivorous and omnivorous/herbivorous species) and a single-rate model, (4) OUM: a two optima, single-rate model, and (5) OUMV: a two optima, two-rate model. This analysis was conducted with SVL data from 119 species (64 insectivorous and 55 omnivorous/herbivorous). For the best-fitting models (equivalent support, ΔAICc ≤ 2), we provide the rate of trait evolution (σ^2), the strength of selection (α), and the phenotypic optimum (θ). We focused on results from the least-complex model (OU1).

**Model**	**ΔAICc**	**Weight**	**Rate of trait evolution (σ^2) and alpha (α)**	**Phenotypic optimum (θ)**
BM1	18.1	<0.001	-	-
BMS	17.5	<0.001	-	-
**OU1**	**0.9**	**0.27**	**σ^2= 0.0014 and α= 0.1 for all species**	**60.6 mm SVL for all species**
OUM	0.6	0.31	σ^2= 0.0014 and α= 0.1 for all species	56.5 mm SVL for insectivorous species and 64.2 mm SVL for omnivorous/herbivorous species
OUMV	0	0.42	σ^2= 0.0016 for insectivorous species, 0.001 for omnivorous species, and α= 0.1 for all species	56.5 mm SVL for insectivorous species and 64.3 mm SVL for omnivorous/herbivorous species

Supplementary Table 6. Summary of five model fits for the body size (snout-to-vent length; SVL) data across 500 randomly-sampled trees from the posterior distribution (one stochastic character map of substrate use for each tree). A brief description of each model is as follows: (1) BM1: a single-rate model of stochastic trait evolution (*i.e.*, rate constrained to be the same for terrestrial and saxicolous species), (2) BMS: a two-rate model of stochastic trait evolution (*i.e.*, separate rates for terrestrial and saxicolous species), (3) OU1: a single phenotypic optimum (*i.e.*, shared optimum for terrestrial and saxicolous species) and a single-rate model, (4) OUM: a two optima, single-rate model, and (5) OUMV: a two optima, two-rate model. This analysis was conducted with SVL data from 123 species (63 terrestrial and 60 saxicolous). For the best-fitting model (OUMV), we provide the rate of trait evolution (σ^2), the strength of selection (α), and the phenotypic optimum (θ).

**Model**	**ΔAICc**	**Weight**	**Rate of trait evolution (σ^2) and alpha (α)**	**Phenotypic optimum (θ)**
BM1	28.7	<0.001	-	-
BMS	18.1	<0.001	-	-
OU1	7.8	0.02	-	-
OUM	5.6	0.06	-	-
**OUMV**	**0**	**0.92**	**$\sigma^{2=}$ 0.0009 for terrestrial species, 0.0019 for saxicolous species, and α= 0.11 for all species**	**58.3 mm SVL for terrestrial species and 66.1 mm SVL for saxicolous species**

Supplementary Table 7. Summary of five model fits for the body size (snout-to-vent length; SVL) data across 500 stochastic character maps of diet, parity mode and substrate use through the maximum clade credibility tree. A brief description of each model is as follows: (1) BM1: a single-rate model of stochastic trait evolution (*i.e.*, rate constrained to be the same for all the species), (2) BMS: a two-rate model of stochastic trait evolution (*i.e.*, separate rates for insectivorous and omnivorous/herbivorous species, for viviparous and oviparous species, or for terrestrial and saxicolous species), (3) OU1: a single phenotypic optimum (*i.e.*, shared optimum for all the species) and a single-rate model, (4) OUM: a two optima (for insectivorous and omnivorous/herbivorous species, for viviparous and oviparous species, or for terrestrial and saxicolous species), single-rate model, and (5) OUMV: a two optima, two-rate model (for insectivorous and omnivorous/herbivorous species, for viviparous and oviparous species, or for terrestrial and saxicolous species). This analysis was conducted with SVL data from 111 species. For the best-fitting models (equivalent support, ΔAICc ≤ 2), we provide the rate of trait evolution (σ^2), the strength of selection (α), and the phenotypic optimum (θ).

**Model**	**Trait**	**AICc**	**ΔAICc**	**Weight**	**Rate of trait evolution (σ^2) and alpha (α)**	**Phenotypic optimum (θ)**
BM1	-	-278.8	17.4	<0.001		
BMS	Diet	-278.1	18.1	<0.001		
	Parity	-277.3	18.9	<0.001		
	Substrate	-283.0	13.2	<0.001		
OU1	-	-290.8	5.4	0.03		
OUM	Diet	-290.7	5.5	0.03		
**OUM**	**Parity**	**-296.2**	**0**	**0.4**	**σ^2=0.0012 and α=0.114 for all species**	**55.6 mm SVL for oviparous species and 67.3 mm SVL for viviparous species**
	Substrate	-292.4	3.8	0.06		
OUMV	Diet	-289.9	6.3	0.02		
	Parity	-294.3	1.9	0.16	σ^2= 0.0013 for oviparous species, 0.0018 for viviparous species, and α=0.1323 for all species	55.8 mm SVL for oviparous species and 66.2 mm SVL for viviparous species
	Substrate	-295.7	0.5	0.31	σ^2= 0.0016 for saxicolous species, 0.0008 for terrestrial species, and α=0.099 for all species	66.1 mm SVL for saxicolous species and 57.1 mm SVL for terrestrial species

Supplementary Table 8. Summary of five model fits for the body size (snout-to-vent length; SVL) data across 500 randomly-sampled trees from the posterior distribution (one stochastic character map of diet, parity mode or substrate use for each tree). A brief description of each model is as follows: (1) BM1: a single-rate model of stochastic trait evolution (*i.e.*, rate constrained to be the same for all the species), (2) BMS: a two-rate model of stochastic trait evolution (*i.e.*, separate rates for insectivorous and omnivorous/herbivorous species, for viviparous and oviparous species, or for terrestrial and saxicolous species), (3) OU1: a single phenotypic optimum (*i.e.*, shared optimum for all the species) and a single-rate model, (4) OUM: a two optima (for insectivorous and omnivorous/herbivorous species, for viviparous and oviparous species, or for terrestrial and saxicolous species), single-rate model, and (5) OUMV: a two optima, two-rate model (for insectivorous and omnivorous/herbivorous species, for viviparous and oviparous species, or for terrestrial and saxicolous species). This analysis was conducted with SVL data from 111 species. For the best-fitting models (equivalent support, ΔAICc ≤ 2), we provide the rate of trait evolution (σ^2), the strength of selection (α), and the phenotypic optimum (θ).

**Model**	**Trait**	**AICc**	**ΔAICc**	**Weight**	**Rate of trait evolution (σ^2) and alpha (α)**	**Phenotypic optimum (θ)**
BM1	-	-272.9	19.5	<0.001		
BMS	Diet	-272.2	20.2	<0.001		
	Parity	-271.9	20.5	<0.001		
	Substrate	-276	16.4	<0.001		
OU1	-	-287.1	5.3	0.03		
OUM	Diet	-287.2	5.2	0.04		
**OUM**	**Parity**	**-292.4**	**0**	**0.49**	**σ^2=0.0016 and α=0.147 for all species**	**55.8 mm SVL for oviparous species and 66.9 mm SVL for viviparous species**
	Substrate	-288.2	4.2	0.06		
OUMV	Diet	-286.2	6.2	0.02		
	Parity	-290.5	1.9	0.19	σ^2= 0.0012 for oviparous species, 0.0013 for viviparous species, and α=0.1176 for all species	55.7 mm SVL for oviparous species and 67 mm SVL for viviparous species
	Substrate	-290.3	2.1	0.17		

Supplementary Table 9. Summary of the effect of parity mode, diet, substrate use, or mean annual temperature on rates of body size evolution using a Bayesian, state-dependent, relaxed-clock model of Brownian motion. Posterior probabilities (PP) that the rates are state-dependent were low (<0.8) in all analyses.

		**Priors**		
		**5-5**	**15-15**	**25-25**
Parity mode<br>*n*= 58 oviparous species and 75 viviparous species	z1 (oviparous)	1.05	0.76	0.84
	z2 (viviparous)	0.95	1.24	1.16
	PP	0.3	0.67	0.51
Diet<br>*n*= 64 insectivorous species and 55 omnivorous/herbivorous species	z1 (insectivorous)	0.85	1.34	1.02
	z2 (omnivorous/herbivorous)	1.15	0.66	0.98
	PP	0.5	0.73	0.23
Substrate use<br>*n*= 63 terrestrial species and 60 saxicolous species	z1 (terrestrial)	0.866	0.99	0.99
	z2 (saxicolous)	0.134	1.01	1.01
	PP	0.48	0.19	0.18

Supplementary Table 10. Path models tested considering the direct (Model 1), indirect (Model 2 to 8), and direct/indirect (Model 9 to 15) effect of viviparity on body size in *Liolaemus* lizards. The direct/indirect Models 15, 13, 14, and 11) and the indirect Model 8 were the best-supported based on the ΔCICc (<2) and CICc weights (w). Further, the not significant two-sided *p*-value (>0.05) of Fisher's C statistic indicates that these models fit the data well. k= number of independent claims, q= number of parameters, and l= relative likelihoods.

**Model**	**k**	**q**	**C**	***p***	**CICc**	**ΔCICc**	**l**	**w**
Model15	3	12	10.35	0.11	37.53	0	1	0.197
Model13	4	11	13.25	0.10	37.92	0.386	0.82	0.163
Model8	4	11	13.51	0.09	38.17	0.644	0.72	0.143
Model14	4	11	13.68	0.09	38.34	0.813	0.67	0.131
Model11	5	10	16.21	0.09	38.41	0.876	0.65	0.127
Model6	5	10	17.79	0.06	39.99	2.46	0.29	0.058
Model12	4	11	15.39	0.05	40.05	2.52	0.28	0.056
Model7	5	10	18.49	0.047	40.69	3.16	0.21	0.04
Model9	5	10	19.05	0.04	41.25	3.72	0.16	0.03
Model4	6	9	22.55	0.03	42.33	4.8	0.09	0.02
Model10	5	10	20.7	0.02	42.9	5.37	0.07	0.01
Model5	5	10	20.95	0.02	43.15	5.62	0.06	0.01
Model1	6	9	24.03	0.02	43.82	6.28	0.04	0.009
Model2	6	9	26.71	0.009	46.49	8.96	0.01	0.002
Model3	6	9	29.87	0.003	49.65	12.12	0.002	>0.001

Supplementary Table 11. Comparison of evolutionary and optimal regressions of body size in response to mean annual temperature (MAT) of *Liolaemus* lizards. Values for phylogenetic half-life ($t_{1/2}$) in millions of years (*Liolaemus* tree length= 26.08 millions of years), stationary variance (V_y), and rate of adaptation (α) for each analysis. O= oviparous, and V= viviparous.

	**Evolutionary**		**Optimal**						
	**Intercept ± *SE***	**Slope ± *SE***	**Intercept ± *SE***	**Slope ± *SE***	$t_{1/2}$	**%**	V_y	**α**	**AICc**
O (n= 58)	1.7933±0.04	-0.003±0.002	1.7933±0.04	-0.0049±0.004	7.26	28	0.005	0.1	-150.8
V (n= 74)	1.8408±0.02	-0.005±0.002	1.8408±0.02	-0.0073±0.003	5.72	22	0.006	0.12	-181.9
All (n= 132)	1.8316±0.02	-0.0048±0.001	1.8316±0.02	-0.0072±0.002	6	23	0.006	0.12	-339.1
