# Supplementary material for: Viviparity imparts a macroevolutionary signature of ecological opportunity in the body size of female Liolaemus lizards
Source: Nat Commun. 2024 Jun 11;15:4966. doi: 10.1038/s41467-024-49464-x (PMC11167029; doi:10.1038/s41467-024-49464-x)
Supplement: Supplementary file 3 — Description of Additional Supplementary Files [file 41467_2024_49464_MOESM3_ESM.pdf]

Description of Additional Supplementary Files.

File name: Supplementary Data 1.

Description: Diet, mean annual air temperature, mean body size of adult females, parity mode, and substrate use of *Liolaemus* lizards. Information comes from previously published data and references for data sources are provided in the same file.

File name: Supplementary Code 1.

Description: Code used to perform the evolutionary analyses. The MuSSCRat analysis was performed in RevBayes (ver. 1.2.1) and the other analyses were performed using R environment for statistical computing (ver. 4.1.1). This Supplementary Code also contains the instructions to install the used software and to run the evolutionary analyses (README file) using specific databases provided for each analysis.
