# Supplementary material for: Viviparity imparts a macroevolutionary signature of ecological opportunity in the body size of female Liolaemus lizards
Source: Nat Commun. 2024 Jun 11;15:4966. doi: 10.1038/s41467-024-49464-x (PMC11167029; doi:10.1038/s41467-024-49464-x)
Supplement: Supplementary file 6 — Reporting Summary [file 41467_2024_49464_MOESM6_ESM.pdf]

Corresponding author(s): Saúl F. Domínguez-Guerrero

Last updated by author(s): May 21, 2024

Reporting Summary

Nature Portfolio wishes to improve the reproducibility of the work that we publish. This form provides structure for consistency and transparency in reporting. For further information on Nature Portfolio policies, see our Editorial Policies and the Editorial Policy Checklist.

Statistics

For all statistical analyses, confirm that the following items are present in the figure legend, table legend, main text, or Methods section.

n/a	Confirmed	
☐	☒	The exact sample size (*n*) for each experimental group/condition, given as a discrete number and unit of measurement
☐	☒	A statement on whether measurements were taken from distinct samples or whether the same sample was measured repeatedly
☐	☒	The statistical test(s) used AND whether they are one- or two-sided<br>*Only common tests should be described solely by name; describe more complex techniques in the Methods section.*
☐	☒	A description of all covariates tested
☐	☒	A description of any assumptions or corrections, such as tests of normality and adjustment for multiple comparisons
☐	☒	A full description of the statistical parameters including central tendency (e.g. means) or other basic estimates (e.g. regression coefficient) AND variation (e.g. standard deviation) or associated estimates of uncertainty (e.g. confidence intervals)
☐	☒	For null hypothesis testing, the test statistic (e.g. F, t, r) with confidence intervals, effect sizes, degrees of freedom and P value noted<br>*Give P values as exact values whenever suitable.*
☐	☒	For Bayesian analysis, information on the choice of priors and Markov chain Monte Carlo settings
☒	☐	For hierarchical and complex designs, identification of the appropriate level for tests and full reporting of outcomes
☒	☐	Estimates of effect sizes (e.g. Cohen's *d*, Pearson's *r*), indicating how they were calculated

Our web collection on statistics for biologists contains articles on many of the points above.

Software and code

Policy information about availability of computer code

Data collection	We did not use software to collect data
Data analysis	We conducted the MuSSCRat analysis in RevBayes (ver. 1.2.1), whereas the other evolutionary analyses were performed using R environment for statistical computing (ver. 4.1.1). To perform the evolutionary analyses in R, we used the next packages: OUwie (ver. 2.6), phytools (ver. 1.0.3), windex (ver. 2.0.3), phylopath (ver. 1.1.3), slouch (ver. 2.1.4).

For manuscripts utilizing custom algorithms or software that are central to the research but not yet described in published literature, software must be made available to editors and reviewers. We strongly encourage code deposition in a community repository (e.g. GitHub). See the Nature Portfolio guidelines for submitting code & software for further information.

Data

Policy information about availability of data

All manuscripts must include a data availability statement. This statement should provide the following information, where applicable:

- Accession codes, unique identifiers, or web links for publicly available datasets
- A description of any restrictions on data availability
- For clinical datasets or third party data, please ensure that the statement adheres to our policy

Information of diet, mean body size of adult females, parity mode, and substrate use of Liolaemus lizards comes from previously published data. That information (including references of data sources), and mean annual air temperature are provided as Supplementary Data 1. Source data are provided with this paper.

Research involving human participants, their data, or biological material

Policy information about studies with human participants or human data. See also policy information about sex, gender (identity/presentation), and sexual orientation and race, ethnicity and racism.

Reporting on sex and gender	*Use the terms sex (biological attribute) and gender (shaped by social and cultural circumstances) carefully in order to avoid confusing both terms. Indicate if findings apply to only one sex or gender; describe whether sex and gender were considered in study design; whether sex and/or gender was determined based on self-reporting or assigned and methods used.* *Provide in the source data disaggregated sex and gender data, where this information has been collected, and if consent has been obtained for sharing of individual-level data; provide overall numbers in this Reporting Summary. Please state if this information has not been collected.* *Report sex- and gender-based analyses where performed, justify reasons for lack of sex- and gender-based analysis.*
Reporting on race, ethnicity, or other socially relevant groupings	*Please specify the socially constructed or socially relevant categorization variable(s) used in your manuscript and explain why they were used. Please note that such variables should not be used as proxies for other socially constructed/relevant variables (for example, race or ethnicity should not be used as a proxy for socioeconomic status).* *Provide clear definitions of the relevant terms used, how they were provided (by the participants/respondents, the researchers, or third parties), and the method(s) used to classify people into the different categories (e.g. self-report, census or administrative data, social media data, etc.)* *Please provide details about how you controlled for confounding variables in your analyses.*
Population characteristics	*Describe the covariate-relevant population characteristics of the human research participants (e.g. age, genotypic information, past and current diagnosis and treatment categories). If you filled out the behavioural & social sciences study design questions and have nothing to add here, write "See above."*
Recruitment	*Describe how participants were recruited. Outline any potential self-selection bias or other biases that may be present and how these are likely to impact results.*
Ethics oversight	*Identify the organization(s) that approved the study protocol.*

Note that full information on the approval of the study protocol must also be provided in the manuscript.

Field-specific reporting

Please select the one below that is the best fit for your research. If you are not sure, read the appropriate sections before making your selection.

☐ Life sciences ☐ Behavioural & social sciences ☒ Ecological, evolutionary & environmental sciences

For a reference copy of the document with all sections, see nature.com/documents/nr-reporting-summary-flat.pdf

Ecological, evolutionary & environmental sciences study design

All studies must disclose on these points even when the disclosure is negative.

Study description	This study focuses on the ecological opportunity (colonization of cold habitats) provided by evolutionary transitions from oviparity (egg-laying) to viviparity (live birth) in female Liolaemus lizards. Using previously published information, we assembled a multi-species dataset on reproductive parity mode, diet, substrate use, mean body size of adult females, and mean annual environmental temperature. We combined our dataset with an ultrametric tree to perform evolutionary analyses and test the association between live birth and rates and patterns of body size evolution. We focused this study on mean body size of adult females because they lay eggs or gave birth to hatchlings, allowing us to connect parity mode, colonization of cool environments, and body size evolution.
Research sample	Our dataset was gathered by previously published data and includes information about parity mode (oviparous or viviparous),diet (insectivorous, herbivorous or omnivorous), substrate use (terrestrial or saxicolous), and mean body size of adult females for 133 Liolaemus species. References of data sources are included in Supplementary Data 1. For the localities where SVL was measured, we gathered data on mean annual temperature (bio1) from the WorldClim database.
Sampling strategy	We conducted a search of mean body size of adult females for every species included in the most recent published phylogeny of Liolaemus. We used two criteria to choose mean body size of females from the literature to build our dataset. 1) We selected papers in which mean SVL from adult females and locality details were provided. 2) When different papers reported mean SVL for the same species, we chose the paper with the highest sample size. For every species for which we found mean body size information, we performed a search for data on parity mode, diet, and substrate use. All the information search was conducted in Google Scholar in English and Spanish.
Data collection	S.F.D.-G., D.E., and C.A.M.-M. recorded all data in an Excel spreadsheet (Supplementary Data 1). All data are accompanied of their respective data sources.
Timing and spatial scale	We assembled our data set between March 2022 and February, 2024.
Data exclusions	No data were excluded of the analyses.

Reproducibility	All evolutionary analyses were performed in triplicate, with qualitatively similar results.
Randomization	We searched for previously published data on Liolaemus lizards, so randomization was not applicable.
Blinding	Blinding was not important to obtain previously published data

Did the study involve field work? ☐ Yes ☒ No

Reporting for specific materials, systems and methods

We require information from authors about some types of materials, experimental systems and methods used in many studies. Here, indicate whether each material, system or method listed is relevant to your study. If you are not sure if a list item applies to your research, read the appropriate section before selecting a response.

Materials & experimental systems

n/a	Involved in the study
☒	☐ Antibodies
☒	☐ Eukaryotic cell lines
☒	☐ Palaeontology and archaeology
☒	☐ Animals and other organisms
☒	☐ Clinical data
☒	☐ Dual use research of concern
☒	☐ Plants

Methods

n/a	Involved in the study
☒	☐ ChIP-seq
☒	☐ Flow cytometry
☒	☐ MRI-based neuroimaging
